# Fracture risk in type 2 diabetic patients: A clinical prediction tool based on a large population-based cohort

**DOI:** 10.1371/journal.pone.0203533

**Published:** 2018-09-07

**Authors:** Daniel Martínez-Laguna, Cristian Tebé, Xavier Nogués, M Kassim Javaid, Cyrus Cooper, Victor Moreno, Adolfo Diez-Perez, Gary S. Collins, Daniel Prieto-Alhambra

**Affiliations:** 1 GREMPAL Research Group, IDIAP Jordi Gol Primary Care Research Institute, Autonomous University of Barcelona, Barcelona, Spain; 2 CIBER of Healthy Ageing and Frailty Research (CIBERFes), Instituto de Salud Carlos III, Majadahonda, Spain; 3 Ambit Barcelona, Primary Care Department, Institut Catala de la Salut, Barcelona, Spain; 4 Biostatistics Unit at Bellvitge Biomedical Research Institute (IDIBELL), L'Hospitalet de Llobregat, Barcelona, Spain; 5 Department of Basic Medical Sciences, Universitat de Barcelona, Barcelona, Spain; 6 Department of Basic Medical Sciences, Universitat Rovira i Virgili, Reus, Tarragona, Spain; 7 Musculoskeletal Research Unit, IMIM-Hospital del Mar, Barcelona, Spain; 8 Oxford National Institute for Health Biomedical Research Centre, University of Oxford, Windmill Road, Oxford, United Kingdom; 9 Medical Research Council (MRC) Lifecourse Epidemiology Unit, University of Southampton, Southampton, United Kingdom; 10 Centre for Statistics in Medicine (CSM), Nuffield Department of Orthopaedics, Rheumatology, and Musculoskeletal Sciences (NDORMS), University of Oxford, Oxford, United Kingdom; 11 Cancer Prevention and Control Program, Catalan Institute of Oncology-IDIBELL, L'Hospitalet de Llobregat, Spain; 12 CIBER Epidemiología y Salud Pública (CIBERESP), Madrid, Spain; Klinikum rechts der Isar der Technischen Universitat Munchen, GERMANY

## Abstract

**Background:**

An increased fracture risk has been described as a complication of Type 2 diabetes mellitus (T2DM). Clinical prediction models for general population have a limited predictive accuracy for fractures in T2DM patients. The aim was to develop and validate a clinical prediction tool for the estimation of 5-year hip and major fracture risk in T2DM patients.

**Methods and results:**

A cohort of newly diagnosed T2DM patients (n = 51,143, aged 50–85, 57% men) was extracted from the Information System for the Development of Research in Primary Care (SIDIAP) database, containing computerized primary care records for >80% of the population of Catalonia, Spain (>6 million people). Patients were followed up from T2DM diagnosis until the earliest of death, transfer out, fracture, or end of study. Cox proportional hazards regression was used to model the 5-year risk of hip and major fracture. Calibration and discrimination were assessed. Hip and major fracture incidence rates were 1.84 [95%CI 1.64 to 2.05] and 7.12 [95%CI 6.72 to 7.53] per 1,000 person-years, respectively. Both hip and major fracture prediction models included age, sex, previous major fracture, statins use, and calcium/vitamin D supplements; previous ischemic heart disease was also included for hip fracture and stroke for major fracture. Discrimination (0.81 for hip and 0.72 for major fracture) and calibration plots support excellent internal validity.

**Conclusions:**

The proposed prediction models have good discrimination and calibration for the estimation of both hip and major fracture risk in incident T2DM patients. These tools incorporate key T2DM macrovascular complications generally available in primary care electronic medical records, as well as more generic fracture risk predictors. Future work will focus on validation of these models in external cohorts.

## Introduction

Type 2 diabetes mellitus (T2DM) is one of the most prevalent long-term comorbidities in the western world, especially in elderly and obese patients. Affecting at least 285 million people worldwide, the number of cases is expected to reach 438 million by the year 2030[1].

An increased fracture risk has been described as a complication of T2DM[2]. Screening or targeting T2DM patients at high fracture risk is a challenge. Bone mineral density (BMD) as measured by dual energy X-ray absorptiometry (DXA) is not sensitive enough[3], and prediction models like FRAX have a limited predictive accuracy for fractures in T2DM patients[4–7]. A few studies have found non-differentiated[8,9] or lower BMD[10] in patients with T2DM, compared to the general population, but the vast majority of studies show a higher/elevated BMD in T2DM as shown by two meta-analyses [11,12].

Disease history (e.g., time from onset, metabolic control) appears to contribute to this association with fracture, as the risk seems to be increased only in individuals with T2DM, whether newly diagnosed[13] or established [14,15], while an impairment in glucose tolerance or fasting glucose is associated with fracture risk lower than or comparable to nondiabetic individuals[15]. Other factors, both skeletal and extra-skeletal, could play a role in the excess risk of fracture observed in T2DM patients. Indeed, T2DM complications such as neuropathy, nephropathy, and visual impairment due to diabetic retinopathy or cataracts are associated with an increased number of falls and related fractures[16–18]. Moreover, changes in bone tissue composition may contribute to a deterioration of bone biomechanical properties[19].

In addition, antidiabetic drugs such as thiazolidinediones have a harmful effect on BMD and fracture risk[20–22] and some studies have associated insulin use with an increased risk of fracture [23,24]. The effect on bone metabolism of some other drugs remains unclear; this includes metformin, sulfonylureas, dipeptidyl peptidase-4 (DPP4) inhibitors, glucagon-like peptide 1 (GLP-1) agonists, and sodium-glucose transport protein2 (SGLT2) inhibitors [25–31]. Moreover, two recent systematic reviews and meta-analysis of epidemiologic studies on the association between T2DM and fracture risk found a significant positive association between T2DM and hip, vertebral, or foot fractures[32,33].

The specific aim of the present study was to develop a clinical prediction model for the estimation of absolute risk of hip and major fracture in T2DM patients. Secondly, we studied the calibration and discrimination performance of the prediction model obtained.

## Materials and methods

### Data source/s

The protocol of the study was approved at the first site by the Research Ethics Committee (REC) of IDIAP Jordi Gol. Data were extracted from the Information System for the Development of Research in Primary Care (Catalan acronym, SIDIAP: www.sidiap.org). The SIDIAP database provides anonymized clinical information as coded by general practitioners in 274 primary care practices in Catalonia, Spain, covering more than 6 million patients (80% of the Catalan population). The representativeness of the SIDIAP database for the overall Catalan population has been reported elsewhere[34].

SIDIAP contains information on socio-demographics, primary care visits, referrals, diagnostic codes using the 10th edition of the International Classification of Diseases (ICD-10), clinical data and immunizations, and other clinical information. SIDIAP is linked to pharmacy invoice data, which provides detailed information on drugs dispensed in community pharmacies under the universal health care system.

SIDIAP data has been previously used to study other aspects related to T2DM assessment and treatment[34] as well as to characterize the epidemiology and describe new predictors of fragility fractures[35–38].

### Participants

Our study cohort consisted of all newly diagnosed T2DM patients aged between 50 and 85 years, identified in SIDIAP database records with a T2DM diagnostic date between 1 January 2006 and 31 December 2013. Patients with any recorded comorbidity that is commonly considered as secondary osteoporosis and those with advanced chronic renal disease (glomerular filtration <15) were not considered and their data were not extracted from the SIDIAP database. The comorbidity exclusion criteria were type I diabetes, osteogenesis imperfecta, hyperthyroidism, hypogonadism or premature menopause, chronic malnutrition or malabsorption, and chronic liver disease. Included participants were followed up from the time of T2DM diagnosis until the earliest of transfer out/migration, fracture, death, or end of study (31 December 2013).

**Fig 1:**
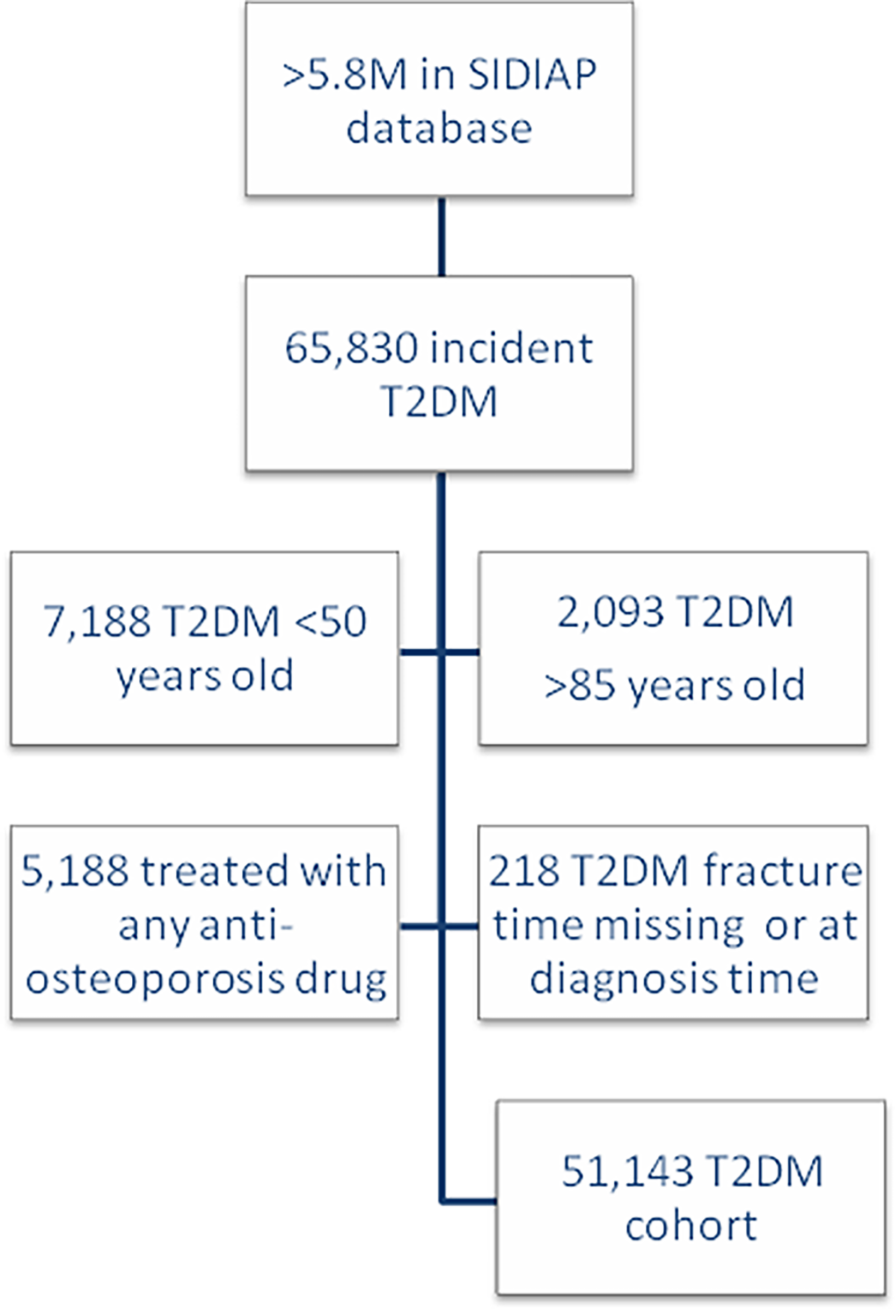
Study flow chart.

Eligible patients treated before the date of T2DM diagnosis with any anti-osteoporosis drug (bisphosphonates, strontium, calcitonin, selective estrogen receptor modulator, and hormone replacement therapy) were excluded. Fig 1 contains a flow chart of the inclusion process.

### Study outcomes

Main outcomes were incident hip fracture and incident major fracture (hip, clinical spine, wrist/forearm, and proximal humerus), identified by previously validated ICD-10 codes[39].

### Potential predictors

The initial set of 35 candidate risk factors to be included in the model was pre-specified based on the literature (i.e., variables potentially associated with increased risk of falls, low BMD, or fragility fractures). These included the following:

1. socio-demographics: age, sex
2. lifestyle factors: body mass index (BMI), alcohol, smoking status
3. laboratory measurements: glycated haemoglobin (HbA1c), and renal function (estimated glomerular filtration rate based on the MDRD4 formula).
4. medications: use of oral corticosteroids for 3 months or more (>5 mg of prednisolone or equivalent), thiazide diuretics, angiotensin-converting enzyme inhibitors, statins, and calcium and vitamin D supplements.
5. co-morbidities: previous record of ischemic heart disease (including angina and myocardial infarction), cerebrovascular disease (CVD), transient ischemic attack, nephropathy, neuropathy, osteoarthritis, cataracts, major fractures, hypoglycemia, and falls.

Interactions were assessed between age and previous major fractures, ischemic disease, nephropathy, and osteoarthritis, and between sex and previous major fractures and oral corticoids. All continuous predictors were assumed to have a linear association with the outcome.

Medications were identified using a pre-specified list of World Health Organization Anatomic Therapeutic Classification (WHO ATC) codes. Co-morbidities were identified using ICD-10 codes. For BMI, HbA1c, estimated glomerular filtration rate (renal function), and smoking status, only results coded within 5 years before the index date were used, selecting the measurement closest to the index date if multiple values were available.

### Statistical analyses

Baseline characteristics of T2DM patients were described using mean and standard deviation for continuous variables and frequencies for categorical variables. Cox regression was used to derive two predictive models for the 5 years following T2DM diagnosis, based on the patient’s medical history and clinical findings: (1) hip fracture risk and (2) major fracture risk.

To develop and validate the clinical prediction model, we followed 7 steps: descriptive analysis, multiple imputations of missing data, bootstrapping, backwards selection of predictors, derivation of a final model, model estimation on imputed data, and models assessment.

First, a descriptive analysis of the assessed factors against outcome was done. Multiple imputation with chained equations (MICE) was then used to minimize the impact of missing data for BMI, HbA1c, serum creatinine (used to calculate renal function), and smoking status. Ten datasets were created, using the Gaussian normal regression method to impute continuous variables (BMI, HbA1c, creatinine) and the multinomial logistic regression method to impute smoking status. Each imputed dataset was sampled by bootstrapping with replacement 100 times, totaling 1,000 samples. Models were fitted for each of the 1,000 samples using backwards elimination (significance level for removal from the model was 0.157). Predictors retained in more than 80% of the 1,000 estimated models were considered for inclusion in the final model. A model with the selected predictors was then estimated using the 10 imputed samples and adjusting coefficients and standard errors for the variability between imputations according to the Rubin rules[40,41]. Finally, discrimination was assessed by estimating Harrell’s C statistics for the final models derived. To assess calibration, observed versus expected fractures were compared graphically by tenths of predicted risk, by 5-year age groups, and by sex. A bootstrapping method[42] was used for internal validation of the estimated models. All data were analyzed using STATA software (StataCorp. 2013. Stata Statistical Software: Release 13. College Station, TX: StataCorp LP) and the Regression Modeling Strategies (rms) package (Frank E Harrell Jr. 2017. R package version 5.1–0.) of R software (R Core Team. 2017. R: A language and environment for statistical computing. R Foundation for Statistical Computing, Vienna, Austria).

## Results

The flow chart of participant inclusion is shown in Fig 1, above. Baseline characteristics of the study participants were stratified by outcome status (Table 1). Patients who sustained a fracture during follow-up were more frequently older, women, systemic steroid users, and had a higher number of associated comorbidities and prevalence of previous fracture/s.

**Table 1:** Baseline patient characteristics according to fracture status [values are numbers (percentages) of patients unless stated otherwise].

	T2DM incident cohort			
	No hip fracture (n = 50,829)	Hip fracture (n = 314)	No major fracture (n = 49,942)	Major fracture (n = 1,201)
**Mean (SD) age (years)**	64.86 (9.28)	74.89 (8.39)	64.79 (9.27)	70.16 (9.63)
**Mean (SD) body mass index (Kg/m**^**2**^**)**	31.11 (5.06)	29.97 (5.10)	31.11 (5.06)	30.89 (5.26)
**Missing body mass index**	20,554 (40.44)	127 (40.32)	20,271 (40.59)	410 (34.14)
**Sex (male)**	29253 (57.55%)	105 (33.44%)	28990 (58.05%)	368 (30.64%)
**Mean (SD) HbA1c**	6.27 (1.32)	6.33 (1.35)	6.27 (1.32)	6.30 (1.38)
**Missing HbA1c**	29458 (57.96%)	169 (53.82%)	29076 (58.22%)	542 (45.13%)
**Smoking status**				
** Never**	9037 (17.78%)	68 (21.66%)	8812 (17.64%)	293 (24.4%)
** Current smoker**	5795 (11.4%)	15 (4.78%)	5741 (11.5%)	69 (5.75%)
** Ex-smoker**	4787 (9.42%)	16 (5.1%)	4729 (9.47%)	74 (6.16%)
** Missing data**	31210 (61.4%)	215 (68.47%)	30660 (61.39%)	765 (63.7%)
**Cerebrovascular disease**	2846 (5.6%)	37 (11.78%)	2777 (5.56%)	106 (8.83%)
**Ischemic heart disease**	4770 (9.38%)	50 (15.92%)	4693 (9.4%)	127 (10.57%)
**Chronic kidney disease**	5076 (9.99%)	71 (22.61%)	4959 (9.93%)	188 (15.65%)
**Neuropathy**	330 (0.65%)	2 (0.64%)	321 (0.64%)	11 (0.92%)
**Cataracts**	4045 (7.96%)	45 (14.33%)	3952 (7.91%)	138 (11.49%)
**History of falling**	611 (1.2%)	10 (3.18%)	588 (1.18%)	33 (2.75%)
**Osteoarthritis**	11250 (22.13%)	103 (32.8%)	10985 (22%)	368 (30.64%)
**Hypoglycemia**	67 (0.13%)	0 (0%)	65 (0.13%)	2 (0.17%)
**Previous hip fracture**	200 (0.39%)	23 (7.32%)	190 (0.38%)	33 (2.75%)
**Previous major fracture**	893 (1.76%)	15 (4.78%)	808 (1.62%)	100 (8.33%)
**Current medications** **Oral steroids for ≥3 months**	3716 (7.31%)	37 (11.78%)	3634 (7.28%)	119 (9.91%)
**Statins**	23697 (46.62%)	135 (42.99%)	23293 (46.64%)	539 (44.88%)
**Thiazides**	6843 (13.46%)	45 (14.33%)	6706 (13.43%)	182 (15.15%)
**Angiotensin enzyme inhibitors**	18523 (36.44%)	128 (40.76%)	18193 (36.43%)	458 (38.13%)
**Calcium and vitamin D**	2729 (5.37%)	32 (10.19%)	2634 (5.27%)	127 (10.57%)

**Fig 2:**
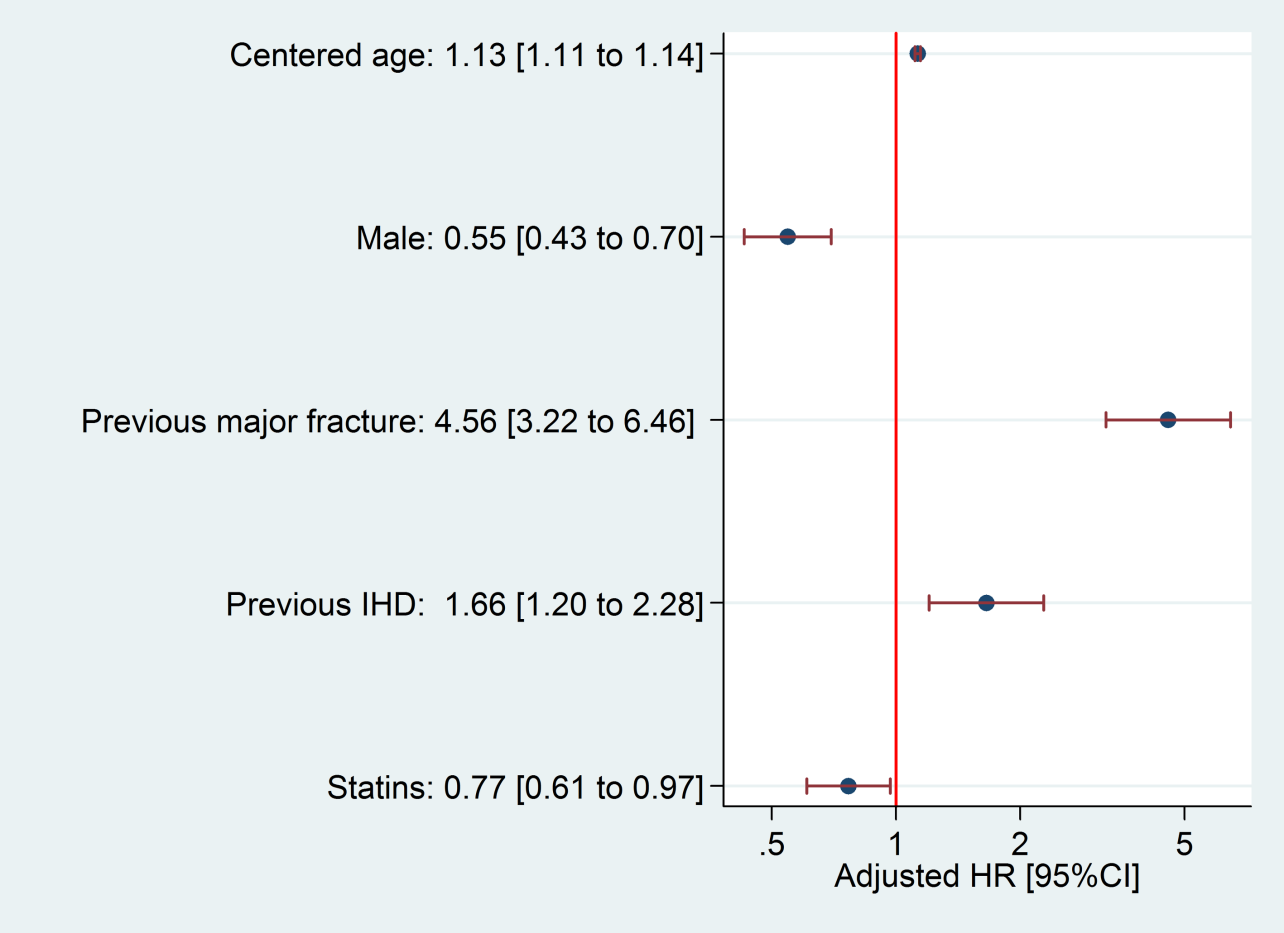
Hazard Ratio and 95% confidence intervals for hip fracture predictors. HR: Hazard Ratio; IHD: Ischemic heart disease.

**Fig 3:**
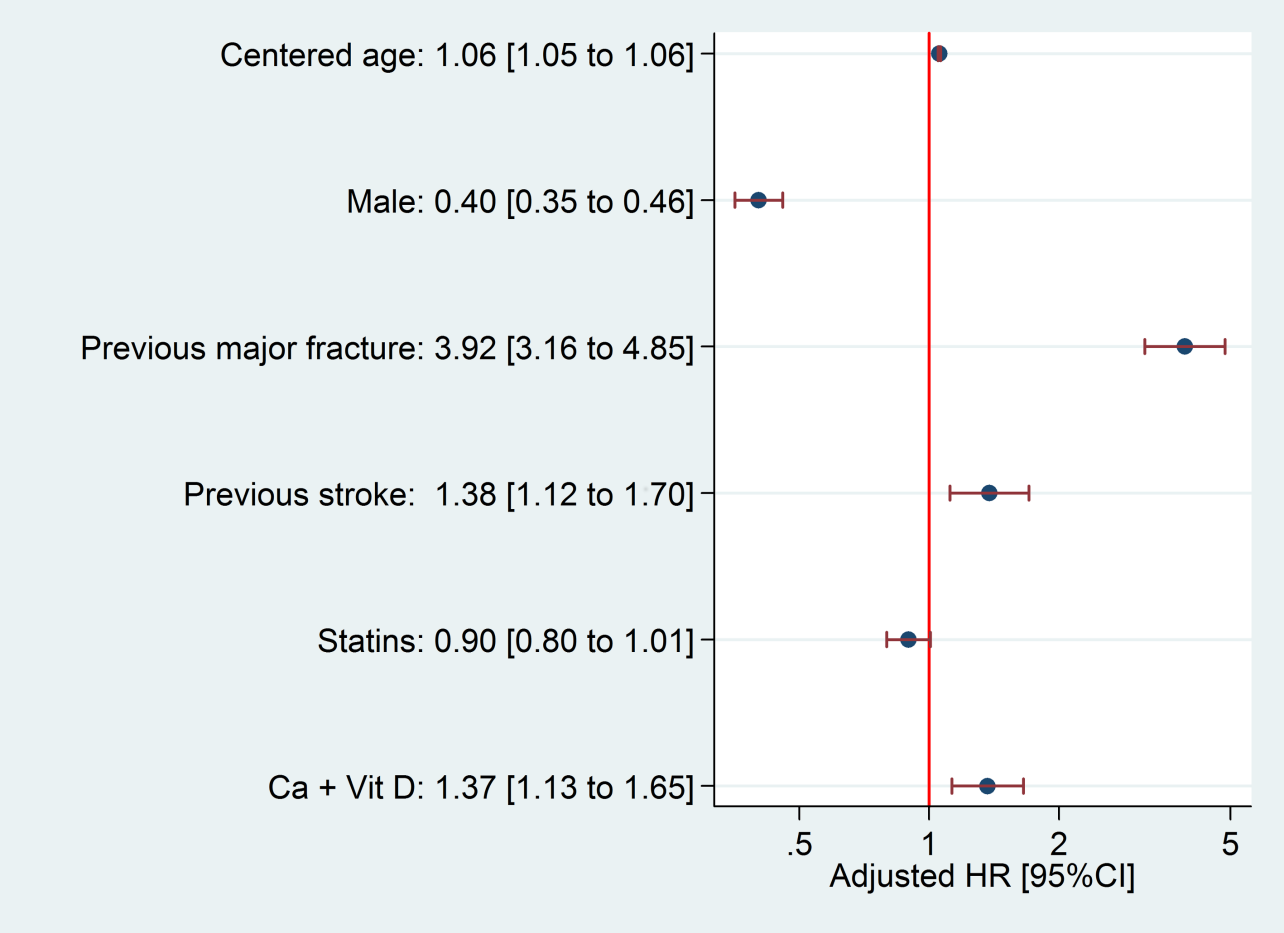
Hazard Ratio and 95% confidence intervals for major fracture predictors. HR: Hazard Ratio; Ca + Vit D: Calcium/vitamin D supplements.

We identified 51,143 newly diagnosed cases of T2DM, observed for a total follow-up of 170,795.3 patient-years for hip fracture, with a median 3.3-year follow-up (interquartile range from 1.7 to 4.9 years). Follow-up for major fracture analysis totaled 168,795.2 patient-years, with a median 3.2-year follow-up (interquartile range from 1.6 to 4.9 years). During follow-up, a total of 314 patients suffered a hip fracture; incidence rate was 1.84 [95%CI 1.64 to 2.05] per 1,000 persons per year. Major fracture occurred in 1,201 patients, an incidence rate of 7.12 [95%CI 6.72 to 7.53] per 1,000 persons per year. Key predictors of hip and major fracture and the proportion of bootstrap models in which they were retained are reported in the S1 Table; HRs for estimated hip and major fracture models are shown in Figs 2 and 3, respectively.

In our cohort, no patient had a combination of factors that predicted a 5-year risk of hip or major fracture greater than >8% or 18%, respectively. Therefore, our data do not allow for accurate prediction above these thresholds, and any extrapolations are of limited reliability. This model could be useful to clinicians as well as researchers to estimate an individual's profile for 5-year risk of developing a hip or major fracture. The web-based calculator would make it easy for physicians to apply our predictive model. The prediction could then be used to identify subjects at high or low risk of fracture so they can be assessed to manage and reduce their risk. Equations and illustrative examples for the calculation of hip and major fracture risk are reported in supplemental files (S1 and S2 Files, respectively) and the online version of both tools is available at https://research.ndorms.ox.ac.uk/fred/. Nonetheless, prediction tools like the ones presented here cannot be used for clinical management until approval has been obtained from regulatory authorities.

**Fig 4:**
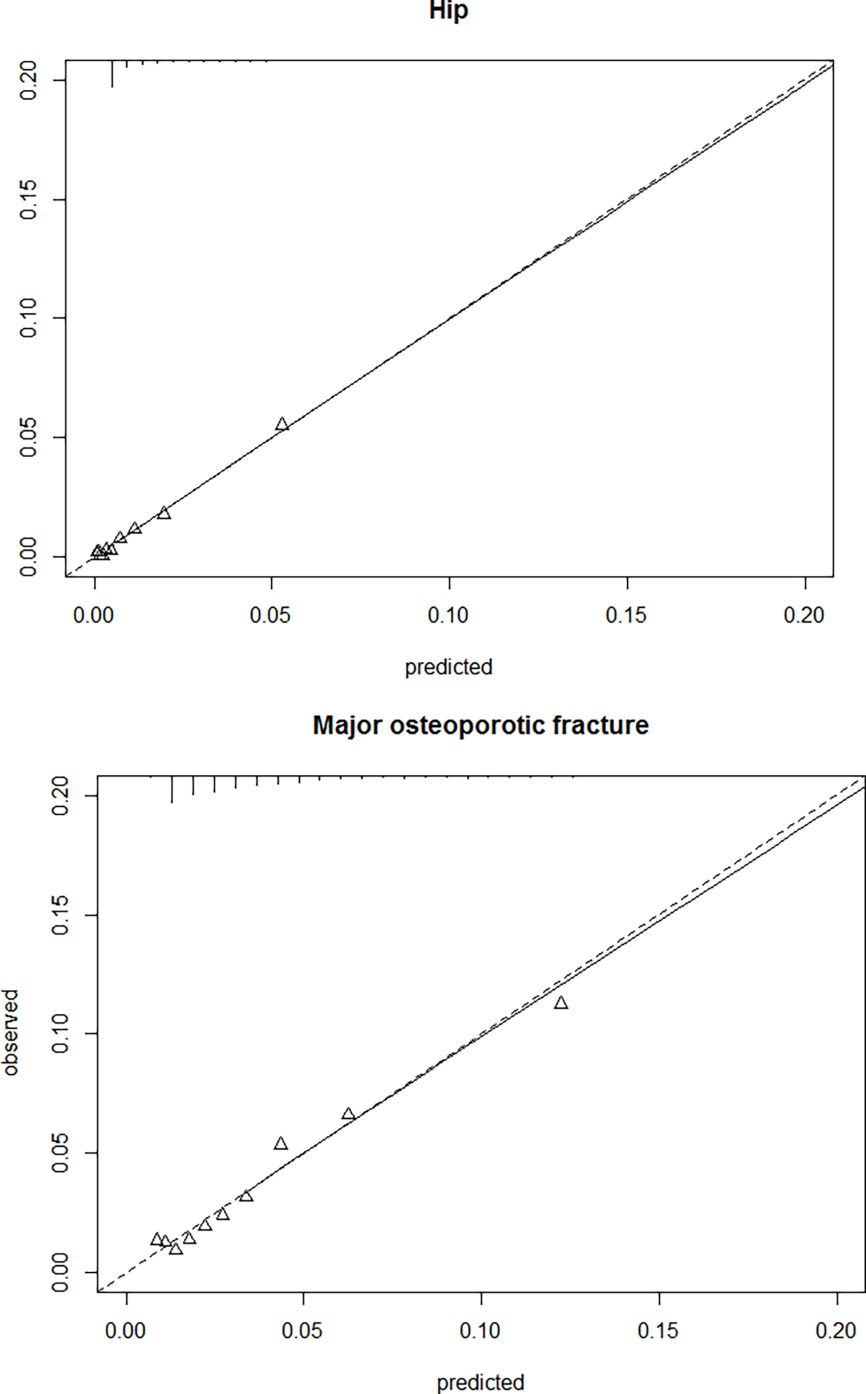
Observed vs predicted risk of hip (top) and major fracture (bottom), stratified by tenths of predicted risk.

**Fig 5:**
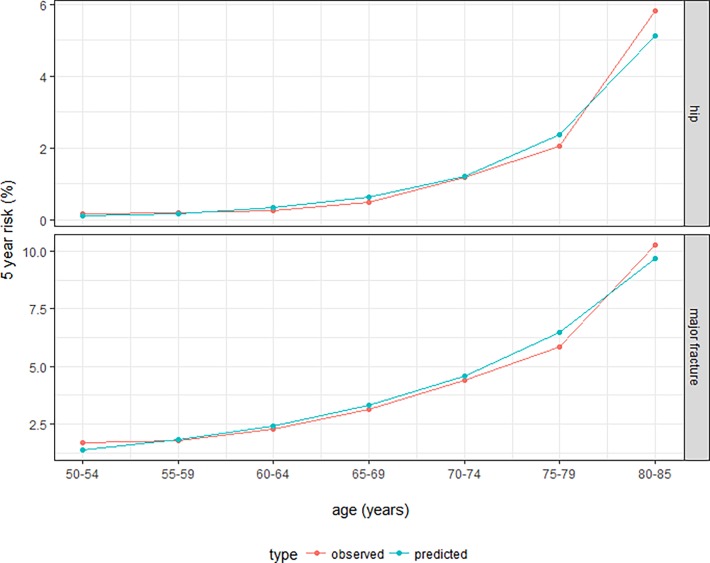
Observed vs predicted risk of hip (top) and major fracture (bottom), stratified by age groups.

The derived clinical prediction model for hip fracture risk had excellent discrimination, with Harrell´s C statistics of 0.81. Calibration showed a good observed/expected ratio for all strata of predicted risk and age/sex combinations (Figs 4 and 5). The proposed model for major fracture prediction also had good discrimination, with Harrell´s C statistic of 0.72, and excellent calibration for all the observed strata (Figs 4 and 5). Internal validation using bootstrap resampling yielded satisfactory results for both models. Corrected Harrell’s C index was 0.81 for the hip model and 0.72 for the major fracture model. Calibration plots illustrated consistent accuracy from the original model and the bootstrap model; the absolute error for 5-year prediction was close to 0.

## Discussion

We describe two prediction models for the estimation of hip and major fracture risk in incident T2DM patients. These models were based on primary care data that are readily available in electronic medical records: age, sex, previous major fracture, diabetic macrovascular complications, use of statins, and previous prescription of calcium and vitamin D supplements. By accounting for T2DM macrovascular complications, these models identified T2DM patients at high risk of fracture more accurately than previous tools derived for the general population that included risk factors for osteoporosis/low bone density.

Age and sex are classic risk factors for osteoporotic fractures, and previous history of fracture confers an increased risk of future fracture/s[43,44]. Our finding of a consistent inverse association with statin use was more surprising, although a 23% reduction in fracture risk amongst users of these drugs was previously reported [45].

In addition, a number of diabetic complications and comorbidities typically clustered in T2DM patients appeared to be predictors of fracture in our models. The association between T2DM and cardiovascular disease is well known[46–48], and increases with time from T2DM onset as well as with poor metabolic control[49]. Previous studies have shown similar associations between fracture and cardiovascular disease, which is included as a predictor in the model used by the QFracture score [50].

The role of vitamin D plus calcium as a risk factor in our major fracture model could be disconcerting. Vitamin D and calcium supplementation is highly associated with a significant reduction in the incidence of non-vertebral fractures[51] and therefore has been used to prevent osteoporotic fractures in patients with evidence or risk of vitamin D and/or calcium insufficiency[52]. Thus, the role of vitamin D and calcium in our model could be a proxy of the severity of risk perceived by the prescriber, as people at higher risk of fracture are more likely to receive these supplements.

Some antidiabetic drugs such as insulin appear to be associated with an increased fracture risk. In our data, this association was not strong enough to include antidiabetic drugs in the final model. In a cohort of prevalent, rather than incident, T2DM patients, antidiabetic drugs likely would have shown a stronger association with fracture risk, as these patients are more likely to be exposed to such medications. Insulin is associated with an increased fracture risk in a number of studies[23,24] but not all[18,53]. On the other hand, non-insulin-dependent patients have increased BMD and lower fracture risk, compared to users of insulin [54].

Increased fracture risk in T2DM patients has been associated, at least in part, with alterations in bone remodeling and bone cell function[55]. Biochemical markers of bone formation such as osteocalcin and telopeptide carboxy-terminal are decreased in T2DM patients[12]. Moreover, sclerostin, a negative regulator of bone formation which competes with the anabolic Wnt β-catenin pathway, is increased in T2DM patients [56] and associated with a higher risk of vertebral fractures[57]. Higher sclerostin levels could reflect the presence of more deeply embedded osteocytes in older bone that has accumulated more microscopic damage. Circulating sclerostin is also increased in T2DM patients with atherosclerotic lesions[58], suggesting a connection with macrovascular complications of diabetes. Finally, bone quality could be conditioned by an increase in advanced glycation endproducts in bone collagen due to a long exposure to hyperglycemia, which would decrease bone resorption and contribute to fragility fractures[59].

### Strengths and limitations

The main limitation of our data is the lack of validation of each individual fracture. However, coding of fractures in SIDIAP has been compared to classical cohort data and hospital databases and shown to be highly specific (>95% for all fracture sites tested) and moderately sensitive (almost 70% for hip fractures)[39]. Also, ICD-10 does not distinguish between traumatic fractures and fragility fractures. A recent study including a random sample of 300 SIDIAP participants aged >50 years with a recorded fracture during 2012 found >90% of hip fractures and >80% of major fractures due to fragility (not related to high-impact trauma)[60].

Another general limitation of our study is the timepoints when lifestyle factors were measured. To minimize misclassification, we only considered information on lifestyle factors as recorded no more than five years before the index date. Smoking status[61], as well as heavy alcohol intake[62], are well-known lifestyle risk factors for fracture. However, these factors were not included in our model because alcohol intake and use of cigarettes are not accurately collected in the SIDIAP database[34], which generates a high number of missing values and a reporting bias.

Important strengths of our data are the high number of patients studied and the robust statistical methods used, including multiple imputation to account for missing data and bootstrapping to minimize overfitting. However, validation in a dataset different from the development sample is required and is anticipated in the near future.

### Conclusions

The proposed prediction models have good discrimination and calibration to estimate the 5-year risk of hip and major fracture in incident T2DM patients. These tools incorporate key T2DM macrovascular complications predictors generally available in primary care electronic medical records, as well as more generic fracture risk predictors. Future work will focus on the validation of our models in external cohorts.

## Supporting information

S1 File Calculation of hip fracture risk in a newly diagnosed T2DM patient. (DOCX)

S2 File Calculation of major fracture risk in a newly diagnosed T2DM patient. (DOCX)

S1 Table Key predictors of hip and major fracture and the proportion of bootstrap models. (DOCX)
