# Supplementary material for: Fracture risk in type 2 diabetic patients: A clinical prediction tool based on a large population-based cohort
Source: PLoS One. 2018 Sep 7;13(9):e0203533. doi: 10.1371/journal.pone.0203533 (PMC6128577; doi:10.1371/journal.pone.0203533)
Supplement: S1 File — (DOCX) [file pone.0203533.s001.docx]

**Supplementary File 1: Calculation of hip fracture risk in a newly diagnosed T2DM patient**

To estimate the probability of developing a hip fracture of a particular patient one should calculate:

$$P\left( hip fracture \right)=1-\hat{S}^{\exp\left( \sum\beta x \right)}$$

where $\hat{S}$ stands for the baseline hip fracture free survival at 5 years (0.9925908 for hip fracture in our cohort); $\left( \sum\beta x \right)$ stands for the linear function of key predictors in the model. Each predictor is multiplied by its corresponding $\beta$ as in the table below.

For illustrative purposes, we calculate here 5-year hip fracture risk for a 65 year old woman with a previous osteoporotic fracture, with no IHD, and who is not taking statins:

| Key factor | beta | Patient | Result |
| --- | --- | --- | --- |
| Centered age | 0.1206772 | 65 | 0.1206772*(65-64.91674)= 0.010047584 |
| Men/ Women | -0.6038511 | No | -0.6038511*0=0 |
| Previous major fracture | 1.517652 | Yes | 1.517652*1=1.517652 |
| Previous IHD | 0.5041638 | No | 0.5041638*0=0 |
| Statins | -0.2647768 | No | -0.2647768*0=0 |
| $\left( \sum\beta x \right)$ | | | 1.52769958 |
| $\exp\left( \sum\beta x \right)$ | | | exp(1.52769958)=4.6075653 |
| $\hat{S}^{\exp\left( \sum\beta x \right)}$ | | | ${0.9925908}^{4.6075653}=$0.96631494 |
| $1-\hat{S}^{\exp\left( \sum\beta x \right)}$ | | | 1-0.96631494=0.03368506 |
| $P\left( hip fracture \right)$ | | | 3.4 |

Based on this, the risk of such a patient sustaining a hip fracture within the next 5 years is **3.4%**.
