# Supplementary material for: Fracture risk in type 2 diabetic patients: A clinical prediction tool based on a large population-based cohort
Source: PLoS One. 2018 Sep 7;13(9):e0203533. doi: 10.1371/journal.pone.0203533 (PMC6128577; doi:10.1371/journal.pone.0203533)
Supplement: S2 File — (DOCX) [file pone.0203533.s002.docx]

**Supplementary File 2: Calculation of major fracture risk in a newly diagnosed T2DM patient**

$$P\left( any osteoporotic fracture \right)=1-\hat{S}^{\exp\left( \sum\beta x \right)}$$

where $\hat{S}$ stands for the baseline major fracture free survival at 5 years (0. 9551388 for major osteoporotic fracture in our cohort); $\left( \sum\beta x \right)$ stands for the linear function of key predictors in the model. Each predictor is multiplied by its corresponding $\beta$.

For an example 60 year old woman with a previous osteoporotic fracture, with no AVC, who is not taking either statins or Calcium/D supplements:

| Key factor | beta | Patient | Result |
| --- | --- | --- | --- |
| Centered age | 0.0545218 | 60 | 0.0545218*(60-64.91674)=-0.26806951 |
| Men/ Women | -0.9094752 | No | -0.9094752*0=0 |
| Previous major fracture | 1.365394 | Yes | 1.365394*1=1.365394 |
| Previous stroke | 0.3226439 | No | 0.3226439*0=0 |
| Statins | -0.1097131 | No | -0.1097131*0=0 |
| Ca + Vit D | 0.3128135 | No | 0.3128135*0=0 |
| $\left( \sum\beta x \right)$ | | | 1.09732449 |
| $\exp\left( \sum\beta x \right)$ | | | exp(1.09732449)=2.99613908 |
| $\hat{S}^{\exp\left( \sum\beta x \right)}$ | | | ${0.9551388}^{2.99613908}=$0.87151813 |
| $1-\hat{S}^{\exp\left( \sum\beta x \right)}$ | | | 1-0.87151813=0.12848187 |
| $P\left( any osteoporoticfracture \right)$ | | | 12.85 |

Based on this, this patient’s estimated 5-year risk of sustaining a major fracture is **12.9%**.
