# Supplementary material for: Fracture risk in type 2 diabetic patients: A clinical prediction tool based on a large population-based cohort
Source: PLoS One. 2018 Sep 7;13(9):e0203533. doi: 10.1371/journal.pone.0203533 (PMC6128577; doi:10.1371/journal.pone.0203533)
Supplement: S1 Table — (DOCX) [file pone.0203533.s003.docx]

Supplementary Table 1: Key predictors of hip and major fracture and the proportion of bootstrap models

|  | HIP FRACTURE | | | MAJOR FRACTURE | | |
| --- | --- | --- | --- | --- | --- | --- |
| Key predictor | n | % | Selected* | N | % | SELECTED* |
| Centered age | 1000 | 100% | Yes | 1000 | 100% | Yes |
| MALE GENDER | 1000 | 100% | Yes | 1000 | 100% | Yes |
| PREVIOUS MAJOR FRACTURE | 1000 | 100% | Yes | 1000 | 100% | Yes |
| PREVIOUS IHD | 1000 | 91% | Yes | 1000 | 73% | No |
| STATINS USE | 1000 | 89% | Yes | 1000 | 82% | Yes |
| INSULIN USE | 1000 | 77% | No | 1000 | 39% | No |
| previous STROKE | 1000 | 72% | No | 1000 | 86% | Yes |
| SEX AND ORAL CORTICOIDS | 1000 | 67% | No | 1000 | 54% | No |
| PREVIOUS NEPHROPATHY | 1000 | 64% | No | 1000 | 27% | No |
| CENTERED BMI | 1000 | 61% | No | 1000 | 57% | No |
| OAD USE | 1000 | 57% | No | 1000 | 37% | No |
| previous osteoarthritis | 1000 | 50% | No | 1000 | 73% | No |
| age and previous ihd | 1000 | 44% | No | 1000 | 41% | No |
| acei use | 1000 | 44% | No | 1000 | 40% | No |
| age and previous nephropathy | 1000 | 39% | No | 1000 | 48% | No |
| age and previous fracture | 1000 | 36% | No | 1000 | 23% | No |
| age and previous osteoarthritits | 1000 | 36% | No | 1000 | 40% | No |
| ex-smoker | 1000 | 34% | No | 1000 | 22% | No |
| smoker | 1000 | 32% | No | 1000 | 24% | No |
| intermediate acting insulin use | 1000 | 32% | No | 1000 | 37% | No |
| oral corticoids use | 1000 | 32% | No | 1000 | 63% | No |
| previous falls | 1000 | 30% | No | 1000 | 72% | No |
| PREVIOUS ANGINA | 1000 | 29% | No | 1000 | 28% | No |
| Thiazolidinediones use | 1000 | 29% | No | 1000 | 28% | No |
| SHORT ACTING INSULIN USE | 1000 | 27% | No | 1000 | 21% | No |
| long acting insulin use | 1000 | 27% | No | 1000 | 32% | No |
| CENTERED HbA1C | 1000 | 25% | No | 1000 | 31% | No |
| mixed insulin use | 1000 | 25% | No | 1000 | 25% | No |
| previous tia | 1000 | 20% | No | 1000 | 18% | No |
| cA+d use | 1000 | 20% | No | 1000 | 88% | Yes |
| previous cataracts | 1000 | 18% | No | 1000 | 15% | No |
| GLINIDES USE | 1000 | 17% | No | 1000 | 20% | No |
| METFORMIN USE | 1000 | 17% | No | 1000 | 69% | No |
| PREVIOUS MYOCARDIAL INFARCTION | 1000 | 15% | No | 1000 | 21% | No |
| inhibitor of alfa-glucosidases USE | 1000 | 14% | No | 1000 | 18% | No |
| PREVIOUS NEUROPATHY | 1000 | 11% | No | 1000 | 55% | No |
| SEX AND PREVIOUS FRACTURE | 1000 | 7% | No | 1000 | 28% | No |
| previous hypoglycaemia | 1000 | 0% | No | 1000 | 0% | No |

OAD = oral antidiabetic drugs; IHD = ischemic heart disease; BMI = body mass index; ACEI = angiotensin-converting enzyme inhibitors; GFR= glomerular filtration rate; CA+D = calcium and vitamin D supplements; TIA = transient ischemic attack
